# New insights into the role of histamine in subventricular zone-olfactory bulb neurogenesis

**DOI:** 10.3389/fnins.2014.00142

**Published:** 2014-06-16

**Authors:** Maria F. Eiriz, Jorge Valero, João O. Malva, Liliana Bernardino

**Affiliations:** ^1^Center for Neuroscience and Cell Biology of Coimbra, University of Coimbra (CNC-UC)Coimbra, Portugal; ^2^Faculty of Medicine, Institute of Biomedical Imaging and Life Sciences (IBILI), University of CoimbraCoimbra, Portugal; ^3^Faculty of Health Sciences, Health Sciences Research Center, University of Beira InteriorCovilhã, Portugal

**Keywords:** subventricular zone, olfactory bulb, neural stem cells, histamine, neurogenesis

## Abstract

The subventricular zone (SVZ) contains neural stem cells (NSCs) that generate new neurons throughout life. Many brain diseases stimulate NSCs proliferation, neuronal differentiation and homing of these newborns cells into damaged regions. However, complete cell replacement has never been fully achieved. Hence, the identification of proneurogenic factors crucial for stem cell-based therapies will have an impact in brain repair. Histamine, a neurotransmitter and immune mediator, has been recently described to modulate proliferation and commitment of NSCs. Histamine levels are increased in the brain parenchyma and at the cerebrospinal fluid (CSF) upon inflammation and brain injury, thus being able to modulate neurogenesis. Herein, we add new data showing that *in vivo* administration of histamine in the lateral ventricles has a potent proneurogenic effect, increasing the production of new neuroblasts in the SVZ that ultimately reach the olfactory bulb (OB). This report emphasizes the multidimensional effects of histamine in the modulation of NSCs dynamics and sheds light into the promising therapeutic role of histamine for brain regenerative medicine.

## Introduction

Brain diseases represent a very demanding worldwide health challenge. Nevertheless, no effective cure exists for the majority of these disorders. The discovery of NSCs in restricted regions of the adult brain redefined it as a plastic organ. Thus, the search for new drug candidates that may enhance stem cells properties and a full knowledge of NSCs biology are crucial to fulfil the actual healthcare and scientific demands.

NSCs reside in two niches of the adult brain: the SVZ lining the lateral ventricles and the subgranular zone (SGZ) in the dentate gyrus (DG) of the hippocampus. Newly born neurons generated in the SGZ migrate short distances toward the granular cell layer, whereas SVZ-derived neuroblasts migrate long distances through the rostral migratory stream (RMS) toward the OB (Eiriz et al., 2011). Interestingly, upon brain injury, some neuroblasts can leave the SVZ/RMS axis to migrate toward damaged areas and differentiate into the specific neuronal/glial phenotype of the injured region. Therefore, a great effort has been taken on the design of stem cells-based strategies to promote brain repair (Ruan et al., 2014). For that purpose it is crucial to identify new factors that can enhance NSCs capabilities to produce new neurons. Herein, we comment on recent data supporting the role of histamine as a robust proneurogenic factor *in vivo* and we also discuss the profits vs. challenges for its usage in stem cell-based brain repair therapies.

### General role of histamine in the central nervous system

Histamine is an amine that has been classically associated with peripheral inflammatory reactions (Dale and Laidlaw, 1910). However, new evidences also highlight its function as a neuromodulator and neuroinflammatory agent. Four receptors mediate the effects driven by histamine: two postsynaptic (H1R, H2R), one presynaptic (H3R), and a forth receptor mainly present in the immune system (H4R). All receptors belong to the family of rhodopsin-like class A receptors coupled to guanine nucleotide-binding proteins (Brown et al., 2001). Neurons, microglia and mast cells are the three cellular reservoirs of histamine in the adult brain (Brown et al., 2001; Katoh et al., 2001). Histaminergic neurons, present in the tuberomammillary nucleus, project numerous ramifications throughout the entire adult brain, allowing histamine to be involved in a broad range of physiological functions, such as sleep-wake control, emotions, learning and memory (Panula and Nuutinen, 2013). Histamine is found at nanomolar levels in the healthy brain (Soya et al., 2008; Croyal et al., 2011; Bourgogne et al., 2012). However, several brain pathological conditions may be associated with an increased degranulation of mast cells in the *choroid plexus*, leading to a massive release of histamine in the CSF and the consequent increase of the blood brain barrier (BBB) permeability. Histaminergic neuronal activity (analyzed by positron emission tomography) was also found to be increased in the lesioned brain parenchyma (Vizuete et al., 2000; Motoki et al., 2005; Yanai and Tashiro, 2007; Kallweit et al., 2013). Importantly, histamine has been described to be involved in several brain pathologies such as seizures (Bhowmik et al., 2012), stroke (Fan et al., 2011), multiple sclerosis (Ballerini et al., 2013; Krementsov et al., 2013), Parkinson and Alzheimer's disease (Shan et al., 2013). Remarkably, histamine may have a dual role and exert either neuroprotective or neurotoxic effects depending on the animal disease model, the receptor/signaling pathway activated and the diversity of histamine and histamine agonists/antagonists administration protocols. A clinically relevant therapeutic platform should take in account all of these distinct criteria, to be successful. Regarding neurogenesis, and although a recent review (Panula et al., 2014) highlights the role of histamine as a stem cell modulator during brain development, very few research studies on the role of histamine as a proneurogenic factor within the postnatal and adult brain were reported.

### Histamine effects on neural stem cell cultures

It is currently clear that histamine is involved in several brain functions but just recently its role as a modulator of stem cell biology has been revealed. We and others have shown that histamine transiently increases intracellular free calcium levels ([Ca^2+^]_i_) in SVZ stem/progenitor cells, embryonic stem cells and carcinoma cells (Tran et al., 2004; Agasse et al., 2008), suggesting the presence of functional histamine receptors in undifferentiated stem/progenitor cells. Particularly, we found that SVZ cells express the three types of histamine receptors, H1R, H2R, and H3R, being H1R the one responsible for the selective increase of [Ca^2+^]_i_ in immature cells (Agasse et al., 2008).

Recently, it was shown that histamine has a strong proneurogenic effect in neonatal SVZ (Bernardino et al., 2012) and in embryonic cortical cell cultures (Molina-Hernández and Velasco, 2008; Rodríguez-Martínez et al., 2012; Molina-Hernández et al., 2013) *via* H1R activation. Histamine may trigger increased transcription of FGFR1 and increased cell proliferation culminating in the differentiation of FOXP2 neuronal cells both *in vitro* and *in vivo* (Rodríguez-Martínez et al., 2012; Molina-Hernández et al., 2013) (Figure S1A). We also showed that histamine induces an increase of the expression of Mash1, Dlx2 and Ngn1 proneurogenic genes and ultimately favors the GABAergic neuronal phenotype. Thus, histamine may be used as an efficient inductor of neuronal differentiation *in vitro* prior NSCs transplantation. In fact, SVZ cells pretreated with poly(lactic-*co*-glycolic) acid (PLGA) microparticles that release histamine succeeded to survive, integrate and differentiate into newly born doublecortin (DCX)-neurons when transplanted into organotypic hippocampal slice cultures and into the DG or striatum of adult mice (Bernardino et al., 2012). Altogether, these data showed that histamine may be a key player in the priming of NSCs toward the neuronal phenotype.

### Role of histamine in the adult SVZ neurogenic niche *in vivo*

**Figure 1:**
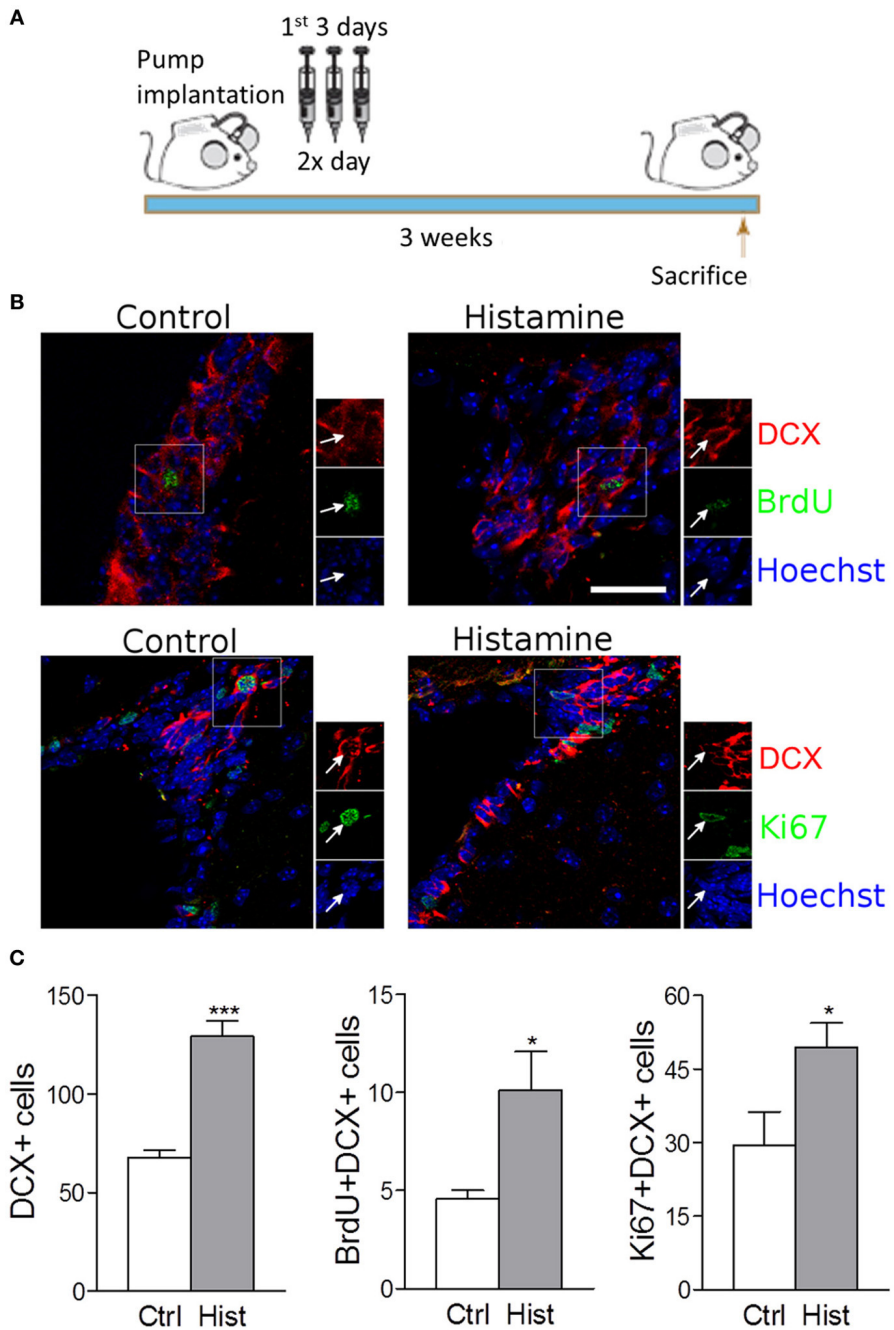
**Intracerebroventricular infusion of histamine triggers neuronal commitment in the SVZ**. **(A)** Design of the 3 weeks experiment, consisting in 3-day BrdU treatment (twice daily with 12 h interval) starting at the day after surgery. Animals were sacrificed 3 weeks after surgery. **(B)** Representative fluorescent confocal digital images of BrdU (green; upper panel), Ki67 (green; lower panel) and DCX (red) positive cells observed in the SVZ of control and histamine treated animals *in vivo*, 21 days after pump installation. Scale bar = 20 μm. Arrows highlight double BrdU+DCX+ (upper panel) or Ki67+DCX+ (lower panel) positive cells. Hoechst staining (blue) labels cell nuclei. **(C)** From left to right: bargraphs represent the total DCX+ positive cells, total BrdU+DCX+ double positive cells and total Ki67+DCX+ double positive cells in both control and histamine *in vivo* treated animals, 21 days after pump installation. Ctrl: Control; Hist: Histamine. Data are expressed as mean ± SEM (*n* = 5–7 mice; ^*^p < 0.05; ^***^p < 0.001). Statistical analysis was performed using Student's unpaired *t*-test.

Despite the absence of *in vivo* studies disclosing the role of histamine in the regulation of the SVZ neurogenic niche, *in vitro* studies have already shown that SVZ NSCs express functional H1R receptors that may be involved in neuronal commitment (Agasse et al., 2008; Bernardino et al., 2012). The relevance of investigating the effects of histamine on SVZ neurogenesis *in vivo* relies on the fact that both inflammation or brain injury may elicit *choroid plexus* mast cells degranulation, increasing the levels of histamine in the CSF and brain parenchyma leading to increased BBB permeability (Anichtchik et al., 2000; Yoshitake et al., 2003; Soya et al., 2008; Kanbayashi et al., 2009; Kallweit et al., 2013). The presence of histamine in the CSF that baths the SVZ neurogenic niche may affect SVZ GFAP-positive stem cells (type B cells) and its progeny *in vivo* by the direct contact of their cilia with the lumen of the lateral ventricles or by the interaction of stem/progenitor cells with the monolayer of ependymal cells (paracrine effect). Interestingly, it was observed that histamine is part of the adult mouse *choroid plexus* transcriptome signature (Marques et al., 2011). Taking into account these considerations, herein we disclose the role of chronic histamine administration in the adult SVZ neurogenic niche *in vivo*. For that, sustained intraventricular infusion of histamine was performed by using mini osmotic pumps that delivered histamine at the CSF for 21 days. All experiments were performed in accordance with the European Community guidelines for the care and use of laboratory animals (86/609/EEC; 2010/63/EU). Weight matched wild-type C57BL/6 8-10 week old male mice were infused at the right lateral ventricle (Anterior-posterior: −0.5 mm, Medial-lateral: 0.7 mm, Dorso-ventral: 3.0 mm) with osmotic mini pumps (Alzet 1004, Charles River, flow rate: 0.10 μ l/h) containing histamine (0.8 mg/Kg, Sigma-Aldrich) dissolved in artificial cerebrospinal fluid (aCSF: 150 mM NaCl, 3 mM KCl, 1.3 mM CaCl_2_, 0.8 mM MgCl_2_, 0.8 mM Na_2_HPO_4_, and 0.2 mM NaH_2_PO_4_) or aCSF alone, as vehicle for 21 days. To ensure stable releasing rates, pumps were incubated before implantation in sterile 0.9% saline at 37°C for 48 h. During the first 3 days after surgery, 50 mg/Kg BrdU was administered intraperitoneally twice a day (Figure 1A). After brain fixation in 4% PFA and cryopreservation in 30% sucrose, 40 μm coronal slices were then cut and stained against Ki67, BrdU, DCX, and NeuN (1:1000 Rabbit polyclonal anti-Ki67—Abcam; 1:1000 Rat monoclonal anti-BrdU—Serotec; 1:1000 Rabbit polyclonal anti-DCX—BD Pharmingen; 1:4000 Mouse monoclonal anti-NeuN—Millipore). Sections were then rinsed and incubated with the appropriate AlexaFluor-conjugated secondary antibodies, stained for Hoechst-33342 and mounted. Confocal digital images were obtained on a LSM 510 Meta; Carl Zeiss microscope. The Software used was Axiovision, release 4.6 (Carl Zeiss) and Image J. Cell counting was performed in confocal images from five slices at 240 μm intervals, both at the SVZ and OB. Only counts performed in the contralateral hemispheres (left) are shown in order to exclude any possible bias induced by inflammatory reactions and/or lesion due to the cannulation at the ipsilateral hemisphere (right).

We found that the intracerebroventricular (i.c.v.) infusion of histamine in the lateral ventricles for 21 days induced a trend increase in the number of BrdU retaining cells (BrdU+DCX−) at the SVZ (Control: 25.8 ± 2.7; Histamine: 34.4 ± 4.7; statistically not-significant). This increase is statistically significant if we consider the BrdU+DCX+ double positive cells (Control: 4.6 ± 0.4; Histamine: 10.1 ± 1.9; p < 0.05; Figure S1B and Figures 1B,C). Accordingly, the number of DCX+ cells increased from 67.6 ± 3.9 in control to 129.1 ± 7.9 in histamine treated mice (*p* < 0.001, Figure 1C). No significant differences were found in counts of BrdU+DCX− and BrdU+DCX+ cells between both ipsilateral and contralateral hemispheres (regarding the same experimental condition) and, most importantly, both hemispheres showed the same relative differences between control and histamine treated animals (data not shown), excluding a putative influence of inflammation and/or tissue damage in the ipsilateral hemisphere. These data confirms previous *in vitro* data identifying histamine as a relevant inductor of neuronal commitment. Curiously, some BrdU+DCX+ cells were retained at the SVZ 21 days upon histamine i.c.v. administration. We may hypothesize that this BrdU+DCX+ cell population at SVZ is derived from BrdU retaining cells, such as quiescent NSCs (B cells) that produce intermediate highly proliferating progenitor cells (C cells). Thus, further studies are also needed to disclose whether this increase of neuroblasts (A cells, BrdU+DCX+ cells) production induced by histamine is due to the activation of B cells which contact with CSF through their apical cilia, or by an increase in the proliferation of C or/and A cells.

Since histamine induced an increase in the number of BrdU+DCX+ cells at the SVZ, we then performed the Ki67 labeling to disclose if histamine had an effect in neuroblast proliferation. Ki67 is a cell marker associated with G1, G2, S and M phases of cell cycle. At the SVZ, Ki67 labelling showed a trend to increase upon histamine treatment (Control: 86.2 ± 24.6; Histamine: 133.0 ± 19.5; statistically not-significant) that was significant when looking to Ki67+DCX+ cells only (Control: 29.4 ± 6.8; Histamine: 49.3 ± 5.0; *p* < 0.05; Figure S1B, and Figures 1B,C). Interestingly, histamine increased the number of BrdU+DCX+ and Ki67+DCX+ cells but did not significantly affected the population of BrdU+DCX− or Ki67+DCX− cells. Altogether, these data indicate that histamine does not induce an overall increase of cell proliferation in the SVZ, but instead may trigger neuronal commitment (as previously showed by us—Bernardino et al., 2012) and/or induce neuroblast proliferation as previously reported (Rodríguez-Martínez et al., 2012; Molina-Hernández et al., 2013).

**Figure 2:**
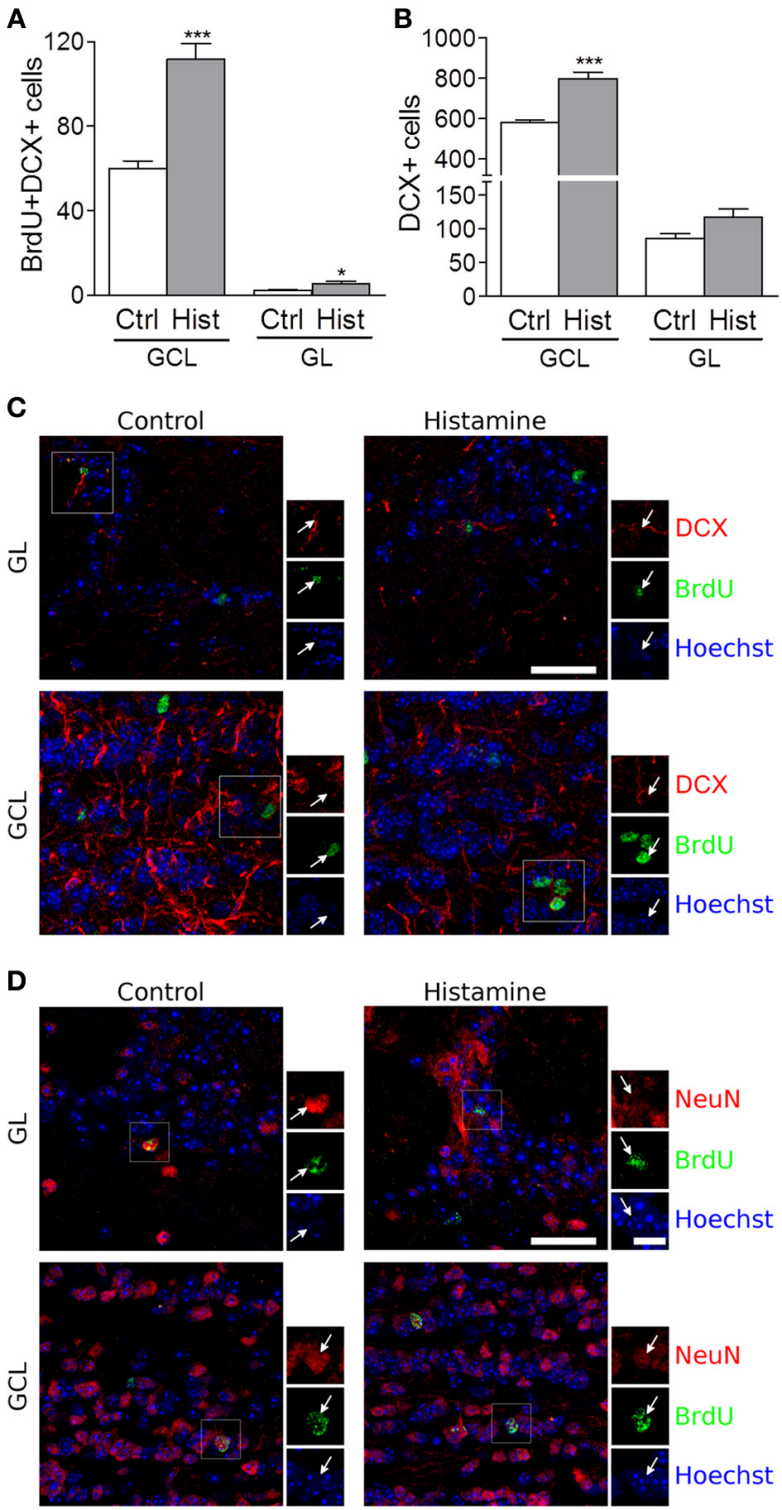
**Olfactory bulb integration of newly differentiated neuroblasts upon histamine long-term treatment**. Bargraphs represent the total BrdU+DCX+ cells **(A)** and total DCX+ positive cells **(B)** counted in the granular cell layer (GCL) and in the glomerular layer (GL). Ctrl, Control; Hist, Histamine. Data are expressed as mean ± SEM (*n* = 5–7 mice; ^*^p < 0.05; ^***^p < 0.001). Statistical analysis was performed using Student's unpaired *t*-test. Representative fluorescent confocal digital images of BrdU (green) and DCX **(**red; **C)** or NeuN **(**red; **D)** positive cells observed in the granule cell layer (GCL) and glomerular layer (GL) of control and histamine treated animals *in vivo*, 21 days after pump installation. White arrows point to double positive BrdU+DCX+ **(C)** or BrdU+NeuN+ **(D)** cells. Hoechst staining (blue) labels cell nuclei. Scale bars = 30 μm.

We also found that SVZ NSCs labelled with BrdU have differentiated into migrating neuroblasts that reached the OB in control and more densely in histamine treated animals (Figure S1B, and Figure 2). An increased number of BrdU+DCX+ cells was found in both the granular cell layer (GCL) and glomerular layers (GL) of the OB (Control GCL: 60.0 ± 3.5; Histamine GCL: 117.7 ± 7.4, *p* < 0.001; Control GL: 2.2 ± 0.2; Histamine GL: 5.5 ± 1.0, *p* < 0.05; Figures 2A,C). A significant increase of the DCX+ cells was also found in the GCL upon histamine infusion (Control GCL: 583.3 ± 11.5; Histamine GCL: 798.4 ± 33.0, *p* < 0.001; Control GL: 86.0 ± 7.1; Histamine GL: 117.3 ± 12.1; Figure 2B). In accordance with the SVZ data, the total number of BrdU+DCX− cells was not significantly different between control and histamine-treated animals in either OB layers (Control GCL: 286.0 ± 29.0; Histamine GCL: 362.6 ± 34.0; Control GL 22.5 ± 5.4; Histamine GL: 33.4 ± 5.3; statistically not-significant). Moreover, Ki67 labeling was almost inexistent at the OB (data not shown). These data may suggest that histamine is not interfering with the overall OB cell proliferation, but, instead, it increases the number of neuroblasts that reach the OB and, therefore, the final population of newly-generated OB neurons (Figure S1B). Additionally, some BrdU+ cells found at GL and GCL of control and histamine-treated animals generated NeuN-mature neurons (Figure 2D).

In fact, histamine is responsible for the priming of NSCs at the SVZ toward the neuronal phenotype, which ultimately will reach the OB. The SVZ-derived progenitor cells are committed to the GCL and GL of the OB, where they differentiate mainly into GABAergic (Bédard and Parent, 2004; De Marchis et al., 2004) but also glutamatergic (Brill et al., 2009) and dopaminergic interneurons (Saino-Saito et al., 2004). Although we do not know the neuronal phenotypes generated by DCX+ neuroblasts found in the OB, we do know that, as expected, the majority of them differentiate into SVZ-derived cells located at the GCL instead of the GL, a structure that mostly contains GABAergic interneurons. Accordingly, we found that histamine induces GABAergic neuronal differentiation in murine SVZ stem cell cultures (Bernardino et al., 2012). Furthermore, the morphology and disposition of DCX+ cells in the GCL and GL of the OB suggest that they are indeed young granule and periglomerular cells (Merkle et al., 2007).

Taken together, our data reveals that histamine is a crucial modulator of neuronal differentiation at the SVZ-OB neurogenic axis. However, we may anticipate some obstacles in using histamine to boost intrinsic regenerative properties of endogenous NSCs. Histamine was also shown to modulate the growth and specification of several cancer types, including gliomas. Increased activity of histidine decarboxylase (HDC), the enzyme involved in histamine synthesis, was found at the surrounding extracellular space of several cancer types, which is suggestive that it may be a crucial factor involved in tumorigenesis. Experiments performed in malignant cell lines and experimental tumors *in vivo* suggest that histamine modulates diverse biological responses related to tumor growth, such as proliferation, survival, and modulation of inflammation and angiogenesis (Eiriz et al., 2014). We could postulate that histamine have the ability to deregulate NSCs dynamics favouring proliferation and boosting the appearance of cancer stem cells especially nearby the neurogenic niches. However, some contradictory reports showed that histamine does not modulate cancer cell proliferation and instead induce their differentiation. Previously, we have shown that histamine does not support proliferation of SVZ stem/progenitor cells *in vitro* (Bernardino et al., 2012). Herein, we showed that the i.c.v. administration of histamine does not induce a significant increase in the total number of BrdU+DCX- or Ki67+DCX- cells at the SVZ, suggesting that, at least after 21 days, histamine does not induce a cancer-like profile of SVZ NSCs cells *in vivo*.

Another limiting factor responsible for the intrinsic difficulties of endogenous brain repair therapies relies on exacerbated inflammatory reactions occurring upon brain lesion that may create a hostile environment for the survival of neural stem/progenitors and neuroblasts. Microglia cells are the main cellular players involved in the innate immune response against brain injury or infection. Microglia phenotypes vary among neurogenic and non-neurogenic regions (Goings et al., 2006) and it may modulate SVZ neurogenesis (Shigemoto-Mogami et al., 2014). In this sense, we recently showed that histamine *per se* stimulates microglia motility and interleukin-1 beta release *via* H4R activation (Ferreira et al., 2012). But, on the other side, in an inflammatory context, histamine inhibited LPS-stimulated microglia activation *via* the same receptor. This dual role of histamine mediating microglial inflammatory responses highlights the need for further studies on the immunomodulatory effects of histamine within neurogenic niches. Increased levels of histamine found upon injury or inflammation can influence the overall cellular micro-environment, including mast cells, ependyma, neurons and microglia, favouring (or not) the survival, proliferation, and differentiation of new cells. This may depend on histamine levels and its distinct actions on different cellular populations present at SVZ-OB neurogenic niches vs. lesioned brain regions.

Recently, Kallweit et al. (2013) have shown that histamine is increased in the CSF of multiple sclerosis patients. In this line, and although we do not show a clear lack of effect of histamine in other neural cell types, such as oligodendrocytes or astrocytes, we have previously shown that histamine did not change NG2 or GFAP expression within the SVZ *in vitro* (Bernardino et al., 2012). Still, more detailed analysis of the effects induced by histamine at the neurogenic niche *in vivo* needs to be accomplished for further therapeutically relevant conclusions.

Upon brain injury, normal cellular dynamics is disturbed and NSCs are de-routed from their quiescent undifferentiated state to an active proliferative state so that new NSCs differentiate into neuroblasts that migrate to the damaged area (Kaneko and Sawamoto, 2009; Grade et al., 2013). Brain repair therapies involving the administration of proneurogenic factors (e.g., histamine) to boost endogenous mobilization of these neuronal precursors is less aggressive than the transplantation of NSCs, but requires a full control of the external booster in order to achieve a fine-tune of the endogenous resources. Alternatively, the transplantation of exogenous stem cells, or stem cell-based progenies at various stages of maturation (e.g., neuroblasts), to replace lost neurons, also raise several limitations, including the possible death of transplanted cells, low number of cells typically available for therapy, inadequate cell differentiation, erroneous cell integration into the host circuitry, immune rejection and variability in the functional outcome of the transplanted cells. Therefore, it is imperative to take in consideration these limitations (endogenous sources vs. exogenous transplantation) to find new effective platforms aiming the repair of damaged brain regions.

Neurogenesis occurring at the SVZ is well documented in rodents, and has also been demonstrated in primates and humans. However, both the cellular organization and the physiologic mechanisms involved on NSCs biology are distinct among these species. The SVZ NSCs found in the human brain younger than 18 months of age actively produce neurons which fate is the OB and the prefrontal cortex (Sanai et al., 2011). However, despite several controversies, a recent report showed that during adulthood human SVZ-derived NSCs lose the ability to migrate toward the OB and, instead, are found in the striatum (Ernst et al., 2014). Importantly, SVZ stem cells extracted from the adult human brain retain the capacities to produce neurons *in vitro*, suggesting that neurogenesis in the SVZ may be boosted under proper stimulation. In fact, several reports showed an increase of striatal neurogenesis in postmortem brains of Huntington disease and stroke patients (Curtis et al., 2003; Macas et al., 2006; Martí-Fàbregas et al., 2010). With our experimental protocol we showed that histamine increased the generation of newly-born neurons at the SVZ that ultimately migrate toward the OB. Thus, differences between human and mouse SVZ niches should be taken in consideration before extrapolating the proneurogenic effect of histamine found in mouse SVZ-OB axis to the potential application in human brain repair strategies. Further studies should disclose whether histamine may also boost neurogenesis under a pathologic condition, such as ischemia, eventually inducing the migration of SVZ-neuroblasts toward the lesioned striatum. Thus, in spite of the potential bottlenecks in triggering an efficient endogenous brain repair, we may asset that histamine efficiently prime NSCs toward the neuronal phenotype, a phenomenon that may support its application in future brain regenerative medicine therapies.

## Author contributions

Maria F. Eiriz: Conception and design; Collection and assembly of data; Data analysis and interpretation; Manuscript writing. Jorge Valero: Conception and design; Collection and assembly of data; Data analysis and interpretation, Manuscript writing. João O. Malva: Conception and design; Provision of study material; Data analysis and interpretation; Administrative support; Critical reading of manuscript. Liliana Bernardino: Conception and design; Provision of study material; Data analysis and interpretation; Financial support; Administrative support; Manuscript writing; Final approval of manuscript.

### Conflict of interest statement

The authors declare that the research was conducted in the absence of any commercial or financial relationships that could be construed as a potential conflict of interest.

## Supplementary material

Figure S1 **Integrative scheme of the effects driven by histamine in stem/progenitor cells both *in vitro* and *in vivo*. (A)** Histamine has been reported to modulate both neuronal differentiation and cell proliferation in diverse types of stem/progenitor cells cultures. Red cells: neurons; Yellow cells: progenitor cells; Blue cell: stem cell. **(B)** Our *in vivo* results showed that histamine increases the number of neuroblasts at the SVZ that reach the OB.
