# Supplementary material for: New insights into the role of histamine in subventricular zone-olfactory bulb neurogenesis
Source: Front Neurosci. 2014 Jun 16;8:142. doi: 10.3389/fnins.2014.00142 (PMC4058902; doi:10.3389/fnins.2014.00142)
Supplement: Figure S1 — Integrative scheme of the effects driven by histamine in stem/progenitor cells both in vitro and in vivo. (A) Histamine has been reported to modulate both neuronal differentiation and cell proliferation in diverse types of stem/progenitor cells cultures. Red cells: neurons; Yellow cells: progenitor cells; Blue cell: stem cell. (B) Our in vivo results showed that histamine increases the number of neuroblasts at the SVZ that reach the OB. [file Presentation1.PDF]

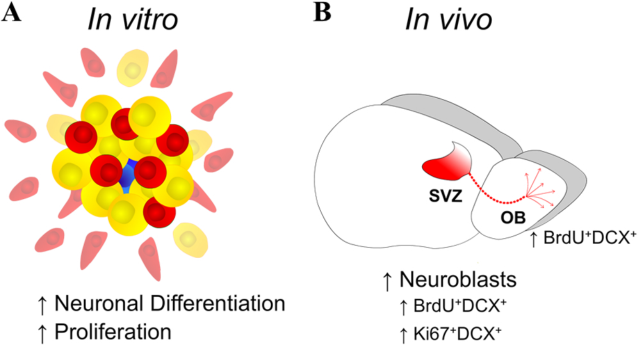

Figure S1: Integrative scheme of the effects driven by histamine in stem/progenitor cells both in vitro and in vivo. (**A**) Histamine has been reported to modulate both neuronal differentiation and cell proliferation in diverse types of stem/progenitor cells cultures. Red cells: neurons; Yellow cells: progenitor cells; Blue cell: stem cell. (**B**) Our in vivo results showed that histamine increases the number of neuroblasts at the SVZ that reach the OB.
